# Risk profile, clinical presentation, and determinants of stroke subtypes among patients with stroke admitted to public referral hospitals, Northwest Ethiopia in 2021: A cross-sectional study

**DOI:** 10.3389/fneur.2022.988677

**Published:** 2022-10-25

**Authors:** Gashaw Walle Ayehu, Getachew Yideg Yitbarek, Edgiet Abebe Zewdie, Bedemariam Tadesse Amsalu, Yonatan Abie, Daniel Atlaw, Assefa Agegnehu, Fitalew Tadele Admasu, Melkalem Mamuye Azanaw, Abraham Tsedalu Amare, Zemen Asmare Emiru

**Affiliations:** ^1^Department of Biomedical Sciences, College of Health Sciences, Debre Tabor University, Debre Tabor, Ethiopia; ^2^School of Medicine, College of Medicine and Health Science, Bahir Dar University, Bahir Dar, Ethiopia; ^3^School of Medicine, College of Health Sciences, Debre Tabor University, Debre Tabor, Ethiopia; ^4^Department of Biomedical sciences, Goba Referral Hospital, Madda Walabu University, Bale-Goba, Ethiopia; ^5^Department of Public Health, College of Health Sciences, Debre Tabor University, Debre Tabor, Ethiopia; ^6^Department of Adult Health Nursing, College of Health Sciences, Debre Tabor University, Debre Tabor, Ethiopia

**Keywords:** risk profile, clinical presentation, determinants, stroke subtype, Ethiopia

## Abstract

**Background:**

Stroke is the second leading cause of death worldwide, with a significant increase in stroke burden over the last two and half decades, especially in developing countries. African countries are undergoing an epidemiological transition from being dominated by infectious diseases to being double-burdened by non-communicable diseases, with existing infectious diseases driven by sociodemographic and lifestyle changes and a weak healthcare system. Data on the risk profile, clinical presentation, and predictors of stroke subtypes are still limited. Therefore, the main aim of this study was to assess the risk profile, clinical presentation, and predictors of stroke in public referral hospitals of Northwest Ethiopia.

**Methods:**

For this study, 554 patients with stroke admitted to three public referral hospitals were prospectively followed up. Data were collected using a pre-tested interviewer-administered questionnaire. STATA version 16 was used for data analyses. Candidate variables significant in bivariate analysis were selected for multivariate binary logistic regression, and statistical significance was set at a *p* < 0.05.

**Results:**

Of the 554 patients with stroke, 60.3% had an ischemic stroke. The mean age of the participants was 61 ± 12.85 years, and more than half (53.25%) of them were women. The most common risk factor identified was hypertension (29.7%), followed by congestive heart failure. The most common clinical presentation was hemiparesis, which was reported by 57.7% of the patients, followed by loss of consciousness (20.7%) and aphasia (9%). Through multivariable logistic regression, age (AOR = 1.03, 95% CI:1.01–1.05), sedentary physical activity level (AOR = 6.78, 95% CI:1.97–23.32), absence of a family history of chronic illness (AOR = 3.79, 95% CI:2.21–6.48), hypertension (AOR=0.51, 95% CI:0.31–0.85), and past stroke (AOR = 3.54, 95% CI:0.93–13.49) were found to be independent determinants of the stroke subtype.

**Conclusion:**

Age, the level of sedentary physical activity, absence of a family history of chronic illness, hypertension, and past stroke were independent determinants of stroke subtype.

## Introduction

Stroke is the second leading cause of death worldwide, with a significant increase in stroke burden over the last two and half decades, especially in developing countries (1). African countries are undergoing an epidemiological transition from being dominated by infectious diseases to being double-burdened by non-communicable diseases, with existing infectious diseases driven by sociodemographic and lifestyle changes and poor healthcare system (2, 3). In 2013, an estimated 535,000 new cases and 2.1 million survivors of stroke were found in Africa (4). Currently, stroke morbidity, mortality, and disability are increasing worldwide (4, 5). Data from the Global Burden of Disease (GBD), Injuries, and Risk Factors study revealed that stroke is the leading cardiovascular disease (CVD) that causes mortality and disability in sub-Saharan Africa (SSA) and other low- and middle-income countries (LMICs) (6).

The prevalence of hemorrhagic stroke (HS) is higher in African countries than in Western countries. This disparity is usually ascribed to racial or genetic factors but may be due to differences in risk factor burden (7), which is largely driven by demographic changes and enhanced by an increasing magnitude of modifiable risk factors (8). Age, sex, family history, and ethnicity are non-modifiable risk factors, while hypertension, diabetes mellites, alcohol, smoking, diet, and physical inactivity are among some of the identified modifiable risk factors (9–13). Underdiagnosis or late diagnosis of hypertension and other risk factors like delayed presentation to the health institution, poor risk factor control, and failure to adhere to treatments are major challenges that need to be addressed (9, 14–17).

The burden, factors, and outcome of stroke vary in Ethiopia between regions and over various time periods (18–22). Risk factors of stroke can be classified into modifiable and non-modifiable factors. Age, sex, family history, and race/ethnicity are non-modifiable risk factors, while renal dysfunction, hyperlipidemia, hypertension, alcohol drinking, smoking, diet, obesity, and physical inactivity are among some of the identified modifiable risk factors (13, 22–24). Stroke can be prevented by lifestyle modification and controlling major risk factors.

Data on risk profiles, clinical presentations, and predictors of stroke subtypes are still limited. Therefore, the main aim of this study was to assess the risk profile, clinical presentation, and predictors of stroke subtypes in public referral hospitals of Northwest Ethiopia.

## Methods

A cross-sectional study was conducted in three public referral hospitals located in Northwest Ethiopia: the University of Gondar Hospital located in Gondar, Tibebe Ghion Specialized Hospital, and Felege Hiwot Regional Referral Hospital located in Bahir Dar. The study was conducted from December to June 2020–2021. The study included 554 patients with stroke (age ≥ 18 years) admitted to and treated at the three public referral hospitals. The inclusion criterion was specified to include all patients with stroke whose diagnosis was confirmed by a CT scan. Patients who were dead before diagnosis confirmation by CT scan, whose initial assessment or diagnosis of stroke was later changed to another case (ruled out stroke) with further evaluation, and who were being treated by other health institutions and referred after treatment or due to complications were excluded.

## Operational definitions

### Diabetes mellitus

Patients who were previously on oral hypoglycemic agents/insulin treatment, who had a diagnosis of any type of DM or an FBS level ≥126 mg/dl, or who had a documented RBS level ≥200 mg/dl or a glycosylated hemoglobin level ≥6.5% (25).

### Dyslipidemia or hyperlipidemia

Patients with a history of hyperlipidemia or using lipid-lowering medication or a total cholesterol level ≥200 mg/dl, an LDL cholesterol level ≥100 mg/ dl, an HDL cholesterol level <40 mg/dl in case of men or <50 mg/dl in case of women, and/or a serum triglyceride level ≥150 mg/dl (26–28).

### Hypertension

Patients receiving antihypertensive medication, who were previously diagnosed with hypertension, or with a blood pressure level ≥140/90 mm/Hg for two measurements (28, 29).

### Alcohol use

Patients consuming ≥2 drinks/day in the case of men and ≥1 drink in the case of women on average (previous drinker/ex-drinker for more than 1 year) (14, 30, 31).

### Smoker

Patients taking two cigarettes per day in the case of men and one per day in the case of women on average (9, 30, 31).

### Former smoker

Patients who abstained from smoking for >1 year (9, 30, 31).

### Current smoker

Patients currently smoking for 1 year (9, 30, 31).

### Physical activity level

It is the measure of planned or incidental activity, including mode or type of activity, frequency of performing an activity, duration of performing an activity, and intensity of performing an activity (32).

### Extremely inactive

It is a PAL < 1.40 (e.g., cerebral palsy) (16, 33).

### Sedentary

Patients with a PAL = 1.40–1.69 (e.g., an office worker with little or no exercise) (16, 33).

### Moderately active

Patients with a PAL = 1.70–1.99 (e.g., construction worker or a person running 1 h daily) (16, 33).

### Vigorously active

Patients with a PAL = 2–2.40 (e.g., agriculture worker non-mechanized or person swimming 2 h daily) (16, 33).

### Extremely active

Patients with a PAL > 2.40 (e.g., competitive cyclist) (16, 33).

## Data collection tool and procedure

Data collection was carried out by an (R-3) internal medicine resident in each hospital with training on the contents of the data collection tool. The data collection tool was developed based on previous research findings and the WHO STEPwise approach to stroke surveillance (34). All relevant data were retrieved from patients' charts, and interviews were conducted with the patients/caregivers using a prepared data extraction form and questionnaire. The patients' history used in the study was obtained from the patients and/or relatives in the language they understood (Amharic). The data collection form was used to collect data on the sociodemographic and behavioral characteristics and clinical characteristics of patients such as risk factors, clinical presentation, and subtypes of stroke.

## Data management and analysis

The collected data were coded, manually checked, and entered into the data management system by using Epi-info version 7. The data were cleaned and edited by simple frequencies and cross-tabulations before analysis, and then, they were exported to STATA version 16 and checked for missing values. Descriptive statistics and numerical summary measures were presented using frequency distribution tables. During candidate selection, adequate significant variables were obtained at a *P* < 0.05, which was considered a cutoff point for multivariate logistic regression analysis using a backward stepwise approach to identify the independent predictors of stroke subtypes. The data were represented as odds ratios (ORs) and 95% confidence intervals.

## Results

The study population comprised 554 patients who were admitted during the study period to the three public referral hospitals, among whom 334 cases (60.29%) had an ischemic stroke and 220 (39.71%) had a hemorrhagic stroke. All the patients were evaluated for stroke using a CT scan.

The mean age of the participants was 61 ± 12.85 years, ranging from 28 to 89 years, and 33% were in the age category of 60–69 years. More than half (53.25%) of the participants were women, and the remaining 46.75% were men. The majority of the participants (68.4%) were rural residents, and 49.64% of patients were housewives. Regarding the educational status of the patients, 67.69% have no formal education. The majority of the patients (84.66%) were married. Regarding behavioral characteristics, 95.49%, 34.3%, and 4.33% consume fruits and vegetables <2 times per week, were former drinkers, and were former smokers, respectively (Table 1).

**Table 1:** Characteristics and risk profile of patients with stroke admitted to public referral hospitals, Northwest Ethiopia, 2020–2021.

**Patient characteristics and stroke risk factor**		**Hemorrhagic stroke** ***N*** **(%)**	**Ischemic stroke** ***N*** **(%)**	**Total** ***N*** **(%)**
Sex	Men	95 (17.1)	164 (29.6)	259 (46.7)
	Women	125 (22.5)	170 (30.7)	295 (53.2)
Age	20–29	5 (0.9)	1(0.18)	6 (1.08)
	30–39	5 (0.9)	15 (2.7)	20 (3.6)
	40–49	30 (5.4)	30 (5.4)	60 (10.8)
	50–59	65 (11.7)	60 (10.8)	125 (22.5)
	60–69	64 (11.5)	120 (21.6)	184 (33.2)
	70–79	45 (8.1)	75 (13.5)	120 (21.6)
	80–89	6 (1.08)	33 (5.9)	39 (7.0)
Residence	Urban	65 (11.7)	110 (19.8)	175 (31.6)
	Rural	155 (28)	224 (40.4)	379 (68.4)
Marital status	Unmarried	5 (0.9)	10 (1.8)	15 (2.7)
	Married	185 (33.4)	284 (51.2)	469 (84.6)
	Divorced	25 (4.5)	10 (1.8)	35 (6.3)
	Widowed	5 (0.9)	30 (5.4)	35 (6.3)
Occupation	Farmer	60 (10.8)	105 (18.9)	165 (29.8)
	Merchant	30 (5.4)	15 (2.7)	45 (8.1)
	Retired	10 (1.8)	25 (4.5)	35 (6.3)
	Government employee	15 (2.7)	19 (3.4)	34 (6.14)
	Housewife	105 (18.9)	170 (30.7)	275 (49.6)
Educational status	No formal education	160 (28.9)	215 (38.8)	375 (67.7)
	Able to read and write	20 (3.6)	65 (11.7)	85 (15.3)
	Primary	10 (1.8)	25 (4.5)	35 (6.3)
	Secondary	10 (1.8)	9 (1.6)	19 (3.4)
	Diploma	10 (1.8)	15 (2.7)	25 (4.5)
	Degree and above	10 (1.8)	5 (0.9)	15 (2.7)
Family history of chronic illness	Yes	40 (7.2)	20 (3.6)	60 (10.8)
	No	179 (32.3)	315 (56.8)	494(89.2)
type of family history of chronic illness	Hypertension	21 (3.8)	10 (1.8)	31 (5.6)
	Diabetes mellitus	10 (1.8)	5 (0.9)	15 (2.7)
	Sudden death	5 (0.9)	0	5 (0.9)
	Ischemic heart disease	5 (0.9)	0	5(0.9)
	Others	0	4 (0.7)	4 (0.7)
Alcohol drinking	Nondrinker	120 (21.6)	185 (33.4)	305 (55)
	Former drinker	70 (12.6)	120 (21.6)	190 (34.3)
	Current drinker	30 (5.4)	29 (5.2)	59 (10.6)
Cigarrate smoking	Nonsmoker	205 (37)	325 (58.6)	530 (95.6)
	Former smoker	15 (2.7)	9 (1.6)	24 (4.3)
	Current smoker	0	0	0
Fruit and vegetable eating	≤2 times per week	215 (38.8)	314 (56.6)	529 (95.5)
	3–4 times per week	5 (0.9)	20 (3.6)	25 (4.5)
Physical activity level	Extremely inactive	10 (1.8)	10 (1.8)	20 (3.6)
	Sedentary	25 (4.5)	70 (12.6)	95 (17.1)
	Moderately active	150 (27.1)	225 (40.6)	375 (67.7)
	Vigorously active	10 (1.8)	5 (0.9)	15 (2.7)
	Extremely active	25 (4.5)	24 (4.3)	49 (8.8)
Presence of comorbidity	Yes	104 (18.8)	165 (29.8)	269 (48.5)
	No	116 (20.9)	169 (30.5)	285 (51.4)
diagnosis of comorbidity	Hypertension	89 (16)	76 (13.7)	165 (29.7)
	Past stroke	5 (0.9)	14 (2.5)	19 (3.4)
	Congestive heart failure	5 (0.9)	30 (5.4)	35 (6.3)
	Diabetes mellitus	0	20 (3.6)	20 (3.6)
	Hyperlipidemia	5 (0.9)	15 (2.7)	20 (3.6)
	Atrial fibrillation	0	5 (0.9)	5 (0.9)
	Migraine headache	0	5 (0.9)	5 (0.9)

### Risk factors for stroke

Table 1 shows the frequency of identified risk factors by stroke subtype. The presence of comorbidity was identified in 269 (48.5%) patients, with 104 patients (47.3%) with HS and 165 patients (49.4%) with IS. The most common comorbidity identified was hypertension, which was in 165 patients (29.7%), followed by CHF in 35 (6.3%), DM in 20 (3.6%), hyperlipidemia in 20 (3.6%), past stroke in 19 (3.4%), and AF in 5 (0.9%). Among patients with hypertension, 89 (40.45%) had a hemorrhagic stroke and 76 (22.6%) had an ischemic stroke.

About 60 (10.8%) patients had a family history of chronic illness, with hypertension being the major chronic illness in the family in 31 patients (5.6%). A total of 190 patients (34.3%) were identified as former drinkers, 59 as current drinkers (10.6%), and the remaining as non-drinkers. Regarding physical activity, a significant share of patients, 95 (17.1%), were sedentary, and 20 (3.6%) were extremely inactive. In total, 529 patients (95.5%) eat fruit and vegetables <2 times per week.

### Clinical presentation of patients with stroke

The most common clinical presentation was hemiparesis as reported by 320 (57.7%) patients, followed by loss of consciousness by 115 patients (20.7%) and aphasia by 50 patients (9%). Most patients with hemorrhage presented with hemiparesis (47.7%), loss of consciousness (34.1%), vomiting (6.8%), and aphasia (6.8%). Similarly, the chief complaint among patients with ischemic stroke was hemiparesis (64.4%), followed by coma (12%), aphasia (10.5%), and slurred speech (6%). Overall, of 225 (40.6%) patients with stroke, 59.1% of patients with hemorrhagic stroke, and 28.4% of patients with ischemic stroke had complications during hospital presentation. Aspiration pneumonia (28.9%) was the most common medical complication, and brain edema (17.1%) was the most common neurologic complication (Table 2).

**Table 2:** Clinical presentation of patients with stroke admitted to public referral public hospitals, Northwest Ethiopia, 2020–2021.

**Clinical presentations**	**Hemorrhagic stroke** ***N*** **(%)**	**Ischemic stroke** ***N*** **(%)**	**Total** ***N*** **(%)**
Coma/loss of consciousness	75 (13.5)	40 (7.2)	115 (20.7)
Vomiting	15 (2.7)	5 (0.9)	20 (3.6)
Dysarthria/ slurred speech	5 (0.9)	20 (3.6)	25 (4.5)
Headache	5 (0.9)	10 (1.8)	15 (2.7)
Facial palsy	0	5 (0.9)	5 (0.9)
Aphasia	15 (2.7)	35 (6.3)	50 (9)
Hemiparesis	105 (18.9)	215 (38.8)	320 (57.7)
Seizure	0	4 (0.7)	4 (0.7)
**Presence of complication**			
Yes	130 (23.4)	95 (17.1)	225 (40.6)
No	90 (16.2)	239 (43.1)	329 (59.4)
Medical complication	110 (19.8)	90 (16.2)	200 (36.1)
Aspiration pneumonia	100 (18)	60 (10.8)	160 (28.9)
DVT	0	10 (1.8)	10 (1.8)
UTI	5 (0.9)	5 (0.9)	10 (1.8)
Electrolyte disturbance	5 (0.9)	10 (1.8)	15 (2.7)
Bedsores/decubitus ulcer	0	5 (0.9)	5 (0.9)
**Neurologic complication**	95 (17.1)	40 (7.2)	135 (24.3)
Increased ICP/brain edema	85 (15.3)	10 (1.8)	95 (17.1)
Herniation	5 (0.9)	10 (1.8)	15 (2.7)
Epileptic seizure	0	10 (1.8)	10 (1.8)
Delirium	5 (0.9)	5 (0.9)	10 (1.8)
Others	0	5 (0.9)	5 (0.9)

### Determinants of stroke subtypes

Through binary logistic regression, age, cigarette smoking, physical activity level, family history of chronic illness, type of comorbidity, complication during admission, and clinical presentation were selected as candidate variables to be included in multivariate logistic regression with a *p* < 0.05. Through multivariate logistic regression, age (AOR = 1.03, 95% CI = 1.01–1.05), the level of sedentary physical activity (AOR = 6.78, 95% CI = 1.97–23.32), absence of a family history of chronic illness (AOR = 3.79, 95% CI = 2.21–6.48), and past stroke (AOR = 3.54, 95% CI = 0.93–13.49) were found to be independent determinants of hemorrhagic stroke subtypes, while hypertension (AOR = 0.51, 95% CI = 0.31–0.85) was independently and strongly associated with an ischemic stroke compared with a hemorrhagic stroke.

With a 1-year increase in patients' age, the odds of having a hemorrhagic stroke compared with ischemic stroke will increase by 3%. Patients with sedentary activity levels were seven times more likely to experience a hemorrhagic stroke than an ischemic stroke. The odds of hemorrhagic stroke vs. ischemic stroke were 3.79 times higher in patients with the absence of a family history of chronic illness than in their counterparts. Furthermore, patients with a history of stroke were 3.54 times more likely to experience a hemorrhagic stroke than an ischemic stroke. Meanwhile, patients with hypertension were 0.51 times less likely to experience a hemorrhagic stroke than an ischemic stroke (Table 3).

**Table 3:** Determinants of stroke subtypes among patients with stroke admitted to public referral hospitals, Northwest Ethiopia, 2020/2021.

**Variables**	**HS**	**IS**	**COR** **(95%, CI)**	**AOR** **(95%, CI)**
Age	220	334	1.02 (1.008–1.035)^***^	1.03 (1.01–1.05)^***^
**Cigarette smoking**				
Non-Smoker	205	321	1	1
Former smoker	15	9	0.37 (0.16–0.88)^*^	0.43 (0.12–1.45)
**Physical activity level**				
Extremely inactive	10	10	1	1
Sedentary	25	70	2.8 (1.04–7.52)^*^	6.78 (1.97–23.32)^**^
Moderately active	150	225	1.5 (0.6–3.69)	4.41 (0.82–12.09)
Vigorously active	10	5	0.5 (0.12–1.99)	0.34 (0.05–2.08)
Extremely active	25	24	0.96 (0.33–2.71)	2.31 (0.69–7.69)
**Family history of chronic illness**				
**Yes**	40	20	1	1
**No**	179	314	3.5(1.98–6.18)^***^	3.18 (1.32–7.68)^**^
**Type of comorbidity**				
No diagnosed comorbidity	116	169	1	1
Hypertension	89	76	0.58 (0.39–0.86)^**^	0.51 (0.31–0.85)^**^
Past stroke	5	14	1.92 (0.67–5.48)	3.54 (0.93–13.49)^*^
CHF	5	30	4.11 (1.55–10.92)^**^	1.75 (0.52–5.91)
Hyperlipidemia	5	15	2.05 (0.72–5.82)	11.18 (0.86–13.23)
**Complication during admission**				
Yes	130	95	1	1
No	90	239	3.63 (2.53–5.20)^***^	3.79 (2.21–6.48)
**Chief compliant**				
Coma/loss of consciousness	75	40	1	1
Vomiting	15	5	0.62 (0.21–1.84)	2.13 (0.62–7.33)
Dysarthria/ slurred speech	5	20	7.5 (2.61–21.48)^***^	3.32 (0.86–12.73)
Headache	5	10	3.75 (1.19–11.72)^*^	0.87 (0.19–3.82)
Aphasia	15	35	4.35 (2.13–8.95)^***^	1.28 (0.5–3.22)
Hemiparesis	105	215	3.83 (2.45–6.01)^***^	1.74 (0.93–3.26)

*** Significant at 0.001 level;

** significant at 0.01 level;

* significant at 0.05 level;

COR, crude odds ratio; AOR, adjusted odds ratio.

## Discussion

The study data were drawn from the large study on stroke conducted in public referral hospitals in Northwest Ethiopia. The findings of this study will lead to advances in and unique contributions to the previously published studies by exploring the risk factors, clinical presentations, and determinant subtypes of stroke.

The mean age of the participants was 61 ± 12.85 years, which was in line with studies conducted in other sub-Saharan African countries (19, 22, 35–37), but it was lower than that in studies conducted in developed countries (38, 39). This disagreement may be due to the Ethiopian population's lifestyle modification, abuse of alcohol, physical inactivity, cigarette smoking, and poor health-seeking behavior, which could result in the early onset of stroke.

In contrast to other previous studies, the percentage of stroke was higher in female patients than in male counterparts (9, 40). The possible reason for the higher percentage among female patients may be due to increased risk factors of stroke such as high use of contraception, pregnancy-related disorders, and migraine.

Almost half of the participants had comorbidity, which include both patients with an ischemic stroke and patients with a hemorrhagic stroke. Similar to other studies conducted in Ethiopia, Senegal, Nigeria, Iran, and China, hypertension was the most common comorbidity (9, 17, 22, 30, 41–43). This trend may reflect that there is still a limitation in screening for hypertension, underdiagnosis, poor blood pressure control in patients with hypertension, and a poor healthcare system.

Considering the habit of alcohol drinking and cigarette smoking, compared with previous studies conducted in Ethiopia, 34.3% former drinkers and 10.6% current drinkers, the habit was low in the current study, with 4.3% former smokers and no current smokers (9, 19). This could be due to underreporting of cigarette smoking, which is seen as being forbidden in the community in the catchment area of our study, although it has high abusers of alcohol. Alcoholism and the risk factor of stroke are associated with aggravating the effects that cause cardioembolism and hypertension.

At hospital presentation, the most common chief complaint was hemiparesis, which was reported by 57.7% of patients, followed by loss of consciousness (20.7%) and slurred speech (4.5%). A similar finding was reported in studies conducted in Ethiopia, India, and Pakistan (8, 35, 41, 44, 45). This finding was unlike other studies from Ethiopia, where a headache was the most common clinical presentation (9). The difference could be because this study was carried out among patients admitted to hospitals, which may contain more severe cases and late hospital presentations, thus leading to disease severity either at onset or in the later stages and complications.

Multivariate logistic regression showed that age, the level of sedentary physical activity, absence of a family history of chronic illness, and past stroke were identified as independent determinants of a hemorrhagic stroke compared with an ischemic stroke, while hypertension was associated with an ischemic stroke compared with a hemorrhagic stroke.

The results of our study revealed that an increase in age is associated with the odds of having a hemorrhagic stroke compared with an ischemic stroke, which is in contrast to studies from Central Africa, Nigeria, and China (7, 23, 46), but supported by a study from Australia (15). However, advanced age with heavy alcohol consumption could predispose individuals to hypertensive intraparenchymal hemorrhage.

Patients with sedentary activity levels were seven times more likely to experience a hemorrhagic stroke than an ischemic stroke (16, 47). The mechanistic basis of the effect of physical activity on stroke risk is likely to prevent obesity, hypertension, dyslipidemia, and the development of type 2 diabetes, all of which are implicated as risk factors of stroke.

The odds of hemorrhagic stroke vs. ischemic stroke were 3.79 times higher in patients with the absence of a family history of chronic illness than in their counterparts, which is supported by different studies (7, 48–50). This could be explained by the fact that a family history of DM, hypertension, and obesity are risk factors for ischemic stroke, largely by their link to atherosclerosis, but the effect of a family history of chronic illness may require further stratification and investigation.

Contradicting a study conducted in Singapore, out study showed that patients with a history of stroke were more likely to experience hemorrhagic stroke than an ischemic stroke (51), which could, in part, be explained by differences in the study population, stroke subtypes, stroke severity, and first-ever or recurrent strokes.

Furthermore, we observed hypertension was independently and strongly associated with ischemic stroke compared with hemorrhagic stroke, which is supported by other studies (7, 48, 49), but this finding contradicts the result of a study conducted in 22 countries (48). A possible explanation may be that, while hypertension predisposes patients to both strokes, atherosclerotic plaque formation (which favors IS) and higher blood pressures may be required for microaneurysmal rupture compared with plaque accident. Finally, in our study, there was no specific clinical presentation significantly associated with the stroke subtypes.

## Strengths and limitations of the study

This study attempted to identify risk factors, clinical presentations, and predictors of stroke subtypes *via* a prospective follow-up of CT scan-confirmed cases in multiple health facilities to ascertain the reliability of the outcome of the study. This may reduce the false-positive and false-negative associations between different variables of the study. The limitation of the study is the study type being a hospital-based study rather than a longitudinal community-based study. Thus, attention should be given to the generalization of the findings to a large community. In addition, we relied on patient reports for some risk factors and other patient-related histories, which may introduce a recall bias.

## Conclusion

The majority of the patients were middle-aged, women, rural residents, and participants with no formal education. The early-onset stroke poses a significant challenge for the healthcare system and the economy of low-income countries. Hypertension was the most common risk factor identified in the current study. The most common clinical presentation was hemiparesis, followed by coma and slurred speech. In addition, the majority of patients with stroke presented with complications during admission.

Age, the level of sedentary physical activity, absence of a family history of chronic illness, hypertension, and past stroke were independent determinants of stroke subtypes. This study has an important clinical implication that uncontrolled hypertension or undiagnosed hypertension are still the targets of wrestling in Ethiopia. Although this strategy in addition to lifestyle modification, screening first-degree relatives for chronic disease and rehabilitation of patients with stroke can be used to reduce the burden of the disease.

## Data availability statement

The raw data supporting the conclusions of this article will be made available by the authors, without undue reservation.

## Ethics statement

The studies involving human participants were reviewed and approved by College of Health Science Ethical Review Committee, Debre Tabor University. The patients/participants provided their written informed consent to participate in this study.

## Author contributions

GA, GY, EZ, ZE, ATA, BA, DA, AA, and MA contributed to the design of the study, analysis, interpretation, and wrote the manuscript. GA, GY, and ZE made the data analysis and interpretation of the data. YA, EZ, DA, AA, FA, and MA contributed to the design of the study, drafting, and edition of the manuscript. All authors critically revised the manuscript and have approved the final manuscript.

## Conflict of interest

The authors declare that the research was conducted in the absence of any commercial or financial relationships that could be construed as a potential conflict of interest.

## Publisher's note

All claims expressed in this article are solely those of the authors and do not necessarily represent those of their affiliated organizations, or those of the publisher, the editors and the reviewers. Any product that may be evaluated in this article, or claim that may be made by its manufacturer, is not guaranteed or endorsed by the publisher.

## Abbreviations

AF: atrial fibrillation
CHF: congestive heart failure
CVD: cardiovascular disease
DM: diabetes mellitus
DVT: deep venous thrombosis
FBS: fasting blood sugar
GBD: global burden of disease
HDL: high-density lipoprotein
HS: hemorrhagic stroke
ICP: intra-cranial pressure
IS: ischemic stroke
LDL: low-density lipoprotein
LMICs: low- and middle-income countries
PAL: physical activity level
RBS: random blood sugar
SSA: sub-Saharan Africa
UTI: urinary tract infection.
